# The effects of the molecular weights of hyaluronic acid on the immune responses

**DOI:** 10.1186/s40824-021-00228-4

**Published:** 2021-08-30

**Authors:** Bo Mi Lee, Sang Jun Park, Insup Noh, Chun-Ho Kim

**Affiliations:** 1 Laboratory of Tissue Engineering, Korea Institute of Radiological and Medical Sciences, 01812 Seoul, Korea; 2 Department of Convergence program of Biomedical Engineering & Biomaterials, The Graduate School, Seoul National University of Science and Technology, Seoul, Korea

**Keywords:** Hyaluronic Acid, GPC, Macrophages, Pro-inflammatory, Anti-inflammatory

## Abstract

**Background:**

The molecular weight of hyaluronic acid (HyA) depends on the type of organ in the body. When HyA of the desired molecular weight is implanted into the human body for regeneration of damaged tissue, it is degraded by hyaluronidase in associated with an inflammatory response. This study sought to evaluate the effects of HyA molecular weight and concentration on pro- and anti-inflammatory responses in murine macrophages.

**Methods:**

The structures and molecular weights of HyAs (LMW-10, MMW-100, MMW-500, and HMW-1,500) were confirmed by ^1^ H NMR and gel permeation chromatography (GPC), respectively. After treatment of murine macrophages with a low (10 µg/mL) or high (100 µg/mL) concentration of each molecular weight HyA, cells were stimulated with lipopolysaccharide (LPS) and changes in immune response in both LPS-stimulated and untreated macrophages were evaluated by assessing nitric oxide (NO) production, and analyzing expression of pro- and anti-inflammatory genes including by RT-PCR.

**Results:**

Molecular weights of LMW-10, MMW-100, MMW-500, and HMW-1,500 were 13,241 ± 161, 96,531 ± 1,167, 512,657 ± 8,545, and 1,249,500 ± 37,477 Da, respectively. NO production by LPS-stimulated macrophages was decreased by increasing concentrations and molecular weights of HyA. At a high concentration of 100 µg/mL, HMW-1,500 reduced NO production in LPS-stimulated macrophages to about 45 %. Using NanoString technology, we also found that the immune-related genes TNF-α, IL-6, IL-1β, TGF-β1, IL-10, IL-11, CCL2, and Arg1 were specifically over-expressed in LPS-stimulated macrophages treated with various molecular weights of HyA. An RT-PCR analysis of gene expression showed that HMW-1,500 decreased expression of classically activated (M1) macrophage genes, such as TNF‐α, IL-6, CCL2, and IL-1β, in LPS-stimulated macrophages, whereas medium molecular-weight HyA (MMW-100 and MMW-500) instead increased expression levels of these genes. HMW-1,500 at a high concentration (100 µg/mL) significantly decreased expression of pro-inflammatory genes in LPS-stimulated macrophages. Expression of genes associated with anti-inflammatory responses (M2 phenotype), such as TGF-β1, IL-10, IL-11, and Arg1, were increased by high concentrations of MMW-500 and HMW-1,500 in LPS-stimulated macrophages.

**Conclusions:**

High molecular-weight HyA (i.e., > 1,250 kDa) inhibits pro-inflammatory responses in LPS-stimulated macrophages and induces anti-inflammatory responses in a concentration dependent manner.

## Background

Hyaluronic acid (HyA) is a linear polymer, composed of regular repeating disaccharide units, β-1, 3-*N*-acetyl-D-glucosamine and β-1, 4-glucuronic acid, is attached to a family of unbranched polysaccharides called glycosaminoglycans (GAGs) [1–3]. HyA is an extracellular matrix component, present in all biological tissues, and body fluids in various molecular sizes [4]. It is found at high levels in the umbilical cord (~ 4 mg/ml; 3.4 × 10^6^ Da) and synovial fluid (3–4 mg/ml; 0.9 × 10^6^ Da), and is also present in the dermis of the skin (~ 0.5 mg/g wet tissue), the epidermis (~ 0.1 mg/g wet tissue), the vitreous of the eye (0.1–0.4 mg/g of wet tissues), and the blood [5]. Based upon other characteristics, such as its good biocompatibility, biodegradability, and viscoelastic properties, HyA is considered an important biomaterial for tissue engineering, drug-delivery systems, and several medical, and pharmaceutical applications [4–6].

The immune system is a defense mechanism comprising many biological structures and processes within an organism that protects against pathogens. The immune system also plays a role in supporting tissue healing [7], which can be invigorated by promoting active acquired immunity to a specific pathogen through vaccine administration [8]. In recent years, biomaterials have also been used for purposes of modulating the immune system, given that HyA polymers are known to be capable of modulating immune responses by interacting with cytokines or macrophages [9].

Immune cells play an important role in maintaining homeostasis. When immune cells detect harmful stimuli such as viral, bacterial or other infections, they respond through an inflammatory response aimed at affected tissues. The goal of this response is to prevent damage to tissue and to return the tissue to its original state [5]. Under homeostatic conditions, production of HyA is balanced by its cellular uptake and degradation [10, 11]. HyA degradation is primarily mediated by members of a family of enzymes called hyaluronidases (HYAL1 and HYAL2) through the CD44 receptor in macrophages [12–14]. HYAL1 and HYAL2 are the major types of hyaluronidases in most tissues and are distinguished by their action at the protein level: HYAL2 is responsible for cleaving high-molecular-weight HyA, which is mostly bound to the CD44 receptor, where HYAL1 degrades HyA in lysosomes to generate HyA oligosaccharides [15].

Inflammation, an immune response that occurs in response to activation of the innate immune system [16], serves to create environmental conditions in the body appropriate for fighting pathogens when body tissues are damaged [7]. When an inflammatory response occurs, immune cells, including monocytes and macrophages, secrete inflammatory mediators such as nitric oxide (NO), tumor necrosis factor (TNF)-α, and interleukin (IL)-6 [17]. Continuing inflammatory responses causes tissue injuries and various diseases, such as arthritis, diabetes, arteriosclerosis, and cancer [18]. Inflammatory responses are achieved through complex mechanisms that alter expression levels of inflammatory genes in response to changes in cell sensitivity and regulation of signaling pathways. During inflammation, high-molecular-weight HyA undergoes a decrease in chain length, most likely owing to hyaluronidase activity [19] or cleavage by reactive oxygen species (ROS) produced in the tissue [20]. HyA uptake and fragmentation by macrophages are thought to be important for resolution of inflammation.

Macrophages, a major cell type in the intrinsic immune system, remove pathogens, and damaged autologous cells from the external environment. Macrophages serve antigen presentation function that contributes to the activation of adaptive immunity. However, inappropriate or chronic activation of macrophages causes excessive damage to normal cells and impairs tissue function, resulting in delayed hypersensitivity, and exacerbation of various autoimmune diseases [21]. Macrophages activate inflammatory responses by recognizing pathogen-associated molecular patterns (PAMPs) of pathogens or damage-associated molecular patterns (DAMPs) generated from damaged tissues [22]. Macrophages are activated by oxidative stress, various cytokines, and bacterial lipopolysaccharides (LPS), the latter of which is a component of gram-negative bacteria that acts as an endotoxin in the body [23], causing a variety of diseases by inducing the production of inflammatory cytokines and NO. Among the receptors for LPS expressed in macrophages are toll-like receptors (TLRs) [24].

TLRs in macrophages are critical components of the innate immune response, responding to a wide range of chemicals produced by bacteria, viruses, and fungi [25]. One type of TLR, TLR-4, can be directly stimulated by LPS and low-molecular-weight HyA, leading to activation of the NF-κB pathway [26]. High-molecular-weight HyA has been shown to prevent LPS activation of macrophages by directly binding to TLR-4.

**Fig. 1:**
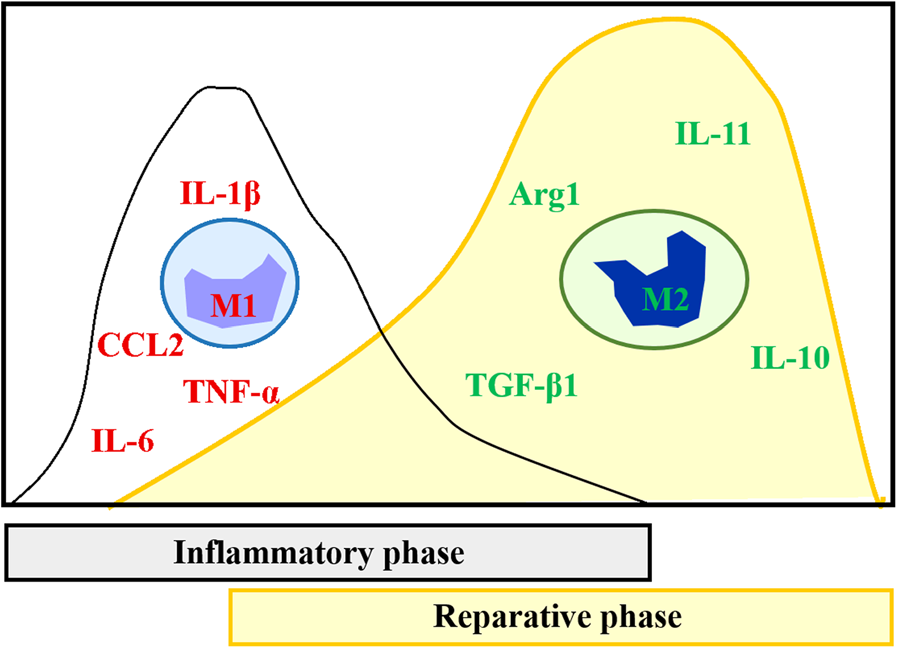
Expression of different immune response factors of macrophages in M1 phase (IL-1β, TNF-α, IL-6, and CCL2) and M2 phase (TGF-β1, IL-10, and IL-11)

There are two types of macrophages at either ends of the broad spectrum of macrophage polarization (Fig. 1): classically activated macrophages, termed M1, alternatively activated macrophages, termed M2 [11]. M1 macrophages are characterized by their ability to induce inflammation and increase the expression of both pro-inflammatory cytokines and chemokines such as IL-1β, IL-6, TNF-α, and C-C motif chemokine ligand 2 (CCL2). M2 macrophages are associated with tissue regeneration and angiogenesis, processes that are also linked to anti-inflammatory cytokines, including tumor growth factor (TGF)-β1, IL-10, and IL-11 [27–29].

The interrelation between the molecular weight and physiological function of HyA can be assessed by monitoring the production of certain inflammatory mediators, which are regulated by the immune system [11, 27]. High-molecular-weight HyA greater than 1,000 kDa exert antiangiogenic, immunosuppressive, and anti-inflammatory effects [11]. These are involved in biological processes such as ovulation, embryogenesis, tissue regeneration and wound repair [27]. High-molecular-weight HyA is also highly viscoelastic and viscous, and can protect cartilage by acting as a lubricant in the synovial fluid. In contrast, medium- and low-molecular-weight HyA possess pro-inflammatory, pro-angiogenic and immunostimulatory properties [30]. However, detailed studies of immune responses caused by HyAs of different molecular weights are currently lacking.

In this study, we explored the effects of various molecular weights (10 to 1,500 kDa) and concentrations (10 and 100 µg/mL) of HyA on pro- and anti-inflammatory responses in LPS-stimulated macrophages. Inflammatory responses of LPS-stimulated macrophages to HyA as a function of HyA molecular weight were assessed by measuring the production of NO released from macrophages. In an effort to more fully understand the immune response systems, we also assessed the effects of various molecular weights of HyA on M1 and M2 polarization with regard to specific pro- and anti-inflammatory gene expression using NanoString and RT-PCR.

## Materials and methods

### ^1^ H nuclear magnetic resonance (^1^ H NMR)

HyAs of Various molecular weight (Sigma-Aldrich, St. Louis, MO, USA) were dissolved in deuterium oxide (D_2_O) at 1.2 wt% at 4℃ overnight. The ^1^ H NMR spectrum was recorded at 25℃ on a 600-MHz spectrometer (Bruker) using 64 scans.

### Gel permeation chromatography (GPC)

For GPC analyses, experiments were carried out at 35 °C using an Aguard Aqueous guard column (50 × 6 mm long; Malvern, Worcestershire, UK) and an A6000M, General Mixed agyeous column (300 × 8 mm long; Malvern) connected in series, at a flow rate of 0.5 mL/min. GPC was performed using a Viscotek GPC/SEC TDAmax detector with refractive index (RI), low-angle light scattering (LALS), right-angle light scattering (RALS), and viscometer systems (Malvern). Samples of HyA with a molecular weight of (0.8 mg/mL) were dissolved in aqueous 0.2 M sodium nitrite containing 0.2 % (w/v) sodium azide and filtered through a syringe filter with a pore size of 0.2 μm (Corning). The molecular weight of HyA in sample (100 µL) was measured by GPC using a refractive increment (dn/dc) of 0.147 ml/g. Poly(ethylene oxide) (PEO; 24 kDa) and dextran (73 kDa, Malvern) were used as standards for calibration of instrument. The results were compiled in OMNISEC Software (Malvern).

### Cell cultures

RAW 264.7 murine macrophages (passage = 26) were obtained from the Korean Cell Line Bank (KCLB). Macrophages were cultured in Dulbecco’s modified Eagle’s medium (DMEM; Gibco) supplemented with 10 % heat-inactivated fetal bovine serum (FBS; Welgene) and a 1 % antibiotic/antimycotic solution (Welgene) at 37℃ in a humidified 5 % CO_2_ incubator.

### NO assay

RAW 264.7 murine macrophages were seeded at a density of 2 × 10^6^ cells/well in 6 well cell culture plates (2 mL/well). After 24 h, the medium was removed, and cells were washed with Dulbecco’s phosphate-buffered saline (DPBS; Welgene). Macrophages were then treated with different concentrations of HyA (10 and 100 µg/mL) for 1 h, after which they were exposed to 100 ng/mL LPS (Sigma-Aldrich, St. Louis, MO, USA) for 24 h. Supernatant media were then collected, and nitrite was measured using the Griess reagent (Promega) according to the manufacturer’s instructions. Briefly, culture supernatants (50 µL) were mixed with 50 µL of the Griess reagent and, after incubating at room temperature for 7 min, their optical density at 540 nm was measured in a microplate reader (Molecular Devices). NO levels were determined directly from standard curves, prepared using serial dilutions of a 100 µM nitrite (50 µL) of the Griess reagent.

### RNA isolation

RNA was isolated using a MasterPure^™^ Complete DNA and RNA purification kit (Lucigen) according to the manufacturer’s instructions. After centrifugation at 10,000 × g for 10 min, supernatants were discarded, and the pellets were re-suspended in 300 µL of tissue and cell lysis solution containing 1 µL proteinase K. After incubation for 15 min at 65 °C, the mixtures were cooled on ice for 5 min and added to 150 µL of MPC protein precipitation reagent. Following centrifugation at 10,000 × g for 10 min, the supernatants were collected and subjected to isopropanol precipitation, after which all residual isopropanol was removed. After an additional treatment with DNase I, total RNA samples were re-suspended in 20 µL of Tris-EDTA (TE) buffer and RNA concentration and quality were assessed using a spectrophotometer (Thermo Fisher Scientific). An A260/A280 ratio between 1.8 and 2.1 indicated optimal RNA purity. RNA was stored at -80 °C until use.

### Measurement of RNA quality and integrity using a bioanalyzer

The quality and integrity of RNA at a concentration of 25 ng/µL was also evaluated using an RNA 6000 Nano kit and 2100 bioanalyzer instrument (Agilent), which provides a measure of the level of degradation, as indicated by RNA integrity number (RIN).

### Gene expression analysis

Gene expression in each sample was assessed using NanoString nCounter Analysis System (NanoString Technologies), as previously described, using a custom-designed codeset containing 280 genes (NanoString Technologies) according to the manufacturer’s instructions. Each reaction contained 150 ng of total RNA, plus reporter and capture probes, together with six pairs of positive control probes and eight pairs of negative control probes. Raw NanoString data were analyzed and normalized using nSolver Analysis Software v4.0 (NanoString Technologies). Counts associated with genes were normalized to internal levels of the reference genes, TNF-α, IL-6, IL-1β, and CCL2 (for M1 macrophages) and TGF-β1, IL-10, IL-11, and Arg1 (for M1 macrophages).

### Reverse transcription polymerase chain reaction (RT-PCR)

cDNA was synthesized from total RNA (1 µg/1 µL) by reverse transcription (RT) in a total volume of 20 µL using an AccuPowerRT preMix kit (BIONEER) following the manufacturer’s instructions. cDNA was stored at -20 °C until use.

PCR reactions were performed in a total volume of 20 µL using a Maxime RT preMix kit (iNtRON Biotechnology) containing 2.5 U i-Star™ Taq DNA polymerase, 10× reaction buffer, gel loading buffer, and 2.5 mM deoxynucleoside triphosphates. The primers (Cosmogenetech) for IL-1β, TNF‐α, IL‐6, CCL2, IL-10, TGF-β1, IL-11, Arg1 and glyceraldehyde‐3‐phosphate dehydrogenase (GAPDH) are listed in Table 1. For each sample, 1 µL of cDNA and each primer at a concentration of 10 pmol/µL with cDNA at 1 µL were added to the reaction mixture, and amplified using the following cycling conditions: initial Samples were subjected to 30 cycles at 94 °C; Initial denaturation for 2 min at 94 °C, denaturation for 20 s at 60 °C, annealing for 10 s at 72 °C, and extension for 40 min, plus a final 72 °C, extension for 3 min. In some experiments, IL-10, IL-11, and Arg1 were subjected to 35 cycles under identical conditions.

**Table. 1:** Genes used for RT-PCR

Gene	Primers (5′→3′)	Accession number
IL-1β	FW: AAATGCCACCTTTTGACAGTGA REV: TCATATGGGTCCGACAGCAC	NM_008361.4
TNF-α	FW: TCAAAATTCGAGTGACAAGCCT REV: TCCAAAGTAGACCTGCCCG	NM_013693.3
IL-6	FW: AGTCCGGAGAGGAGACTTCA REV: TTGGAAATTGGGGTAGGAAGGA	NM_031168.2
CCL2	FW: CCCAATGAGTAGGCTGGAGAG REV: CTGTCACACTGGTCACTCCTAC	NM_011333.3
IL-11	FW: CTGACGGAGATCACAGTCTGGA REV:GGACATCAAGTCTACTCGAAGCC	NM_008350.4
TGF-β1	FW: ATGCTAAAGAGGTCACCCGC REV:TTCCGTCTCCTTGGTTCAGC	NM_011577.2
IL-10	FW: GCTGGACAACATACTGCTAACC REV: ATTTCCGATAAGGCTTGGCAA	NM_010548.2
Arg1	FW: CATTGGCTTGCGAGACGTAGAC REV: GCTGAAGGTCTCTTCCATCACC	NM_007482.3
GAPDH	FW: TCAACGACCCCTTCATTGAC REV:ATGCAGGGATGATGTTCTGG	NM_008084.3

### Statistical analysis

All data are presented as means ± S.D. of nine independent experiments for each test. Each experiment was performed in quadruplicate to ensure reproducibility. *P*-values < 0.01 were considered statistically significant; individual P-values by asterisks (*p < 0.05, **p < 0.01, and ***p < 0.001) in figure legends.

## Results

### Chemical properties of HyAs

To explore the effects of different molecular weight forms of HyA on inflammatory response in LPS-unstimulated and -stimulated macrophages, we used commercial HyA with nominal molecular weights of 10 kDa, 100 kDa, 500 kDa and 1,500 kDa, designated LMW-10, MMW-100, MMW-500 and HMW-1,500, respectively. First, we evaluated the structures and actual molecular weights of each HyA by ^1^ H NMR and GPC, respectively.

**Fig. 2:**
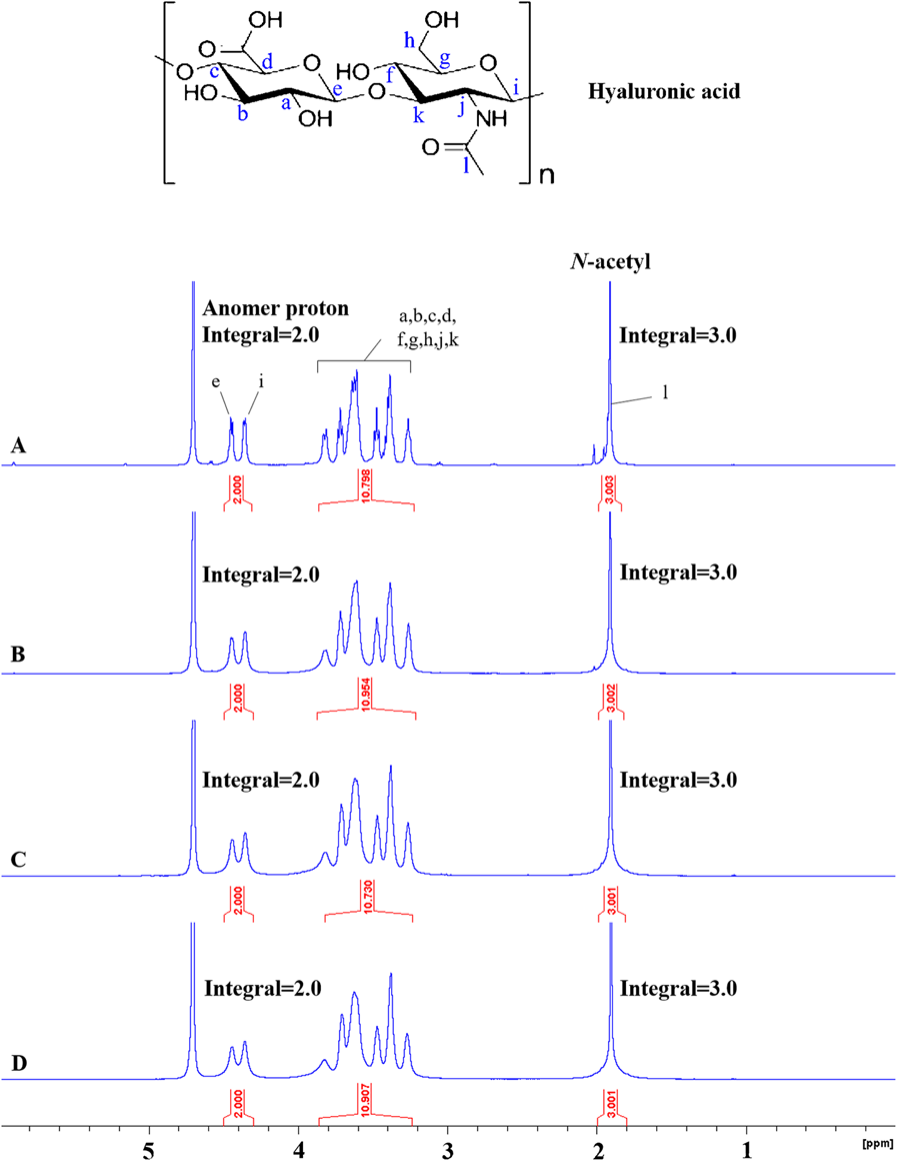
^1^ H NMR spectra of HyA with different molecular weights. The ^1^ H-NMR spectra was recorded using a Bruker Ascend^™^ 600-MHz spectrometer, where **A** LMW-10: low molecular weight of HyA (10 kDa), **B** MMW-100: medium molecular weight of HyA (100 kDa), **C** MMW-500: medium molecular weight of HyA (500 kDa), **D** HMW-1,500: high molecular weight of HyA (1,500 kDa)

HyA is the repeating unit of a GAG composed of β-1, 3-*N*-acetyl-*D*-glucosamine and β-1, 4-glucuronic acid (Fig. 2) [28, 30]. As shown in ^1^ H NMR spectra, there was a broad signal between 3.2 and 3.9 ppm in the spectrum of HyAs of various molecular weights that corresponded to protons in the sugar rings (Fig. 2 A-D, labels a, b, c, d, f, g, h, and k) [31]. Although signals overlapped, making it difficult to assign each proton individually, it was possible to assign signals according to their corresponding protons in the sugar rings. We also detected a characteristic signal between 4.3 and 4.5 ppm corresponding the two anomeric protons attached to the carbons adjacent to the two oxygen atoms (Fig. 2 A-D, labels e and i). The methyl protons of the *N*-acetyl group of HyA showed a signal between 1.8 and 2.0 ppm (Fig. 2 A-D, label l), and the D_2_O peak showed a signal between 4.6 and 4.8 ppm. Integration of LMW-10, MMW-100, MMW-500 and HMW-1,500 revealed a proton ratio of 2.0 (anomeric protons):10.0 (protons of the sugar rings):3.0 (protons of the methyl group). We confirmed that chemical structure of HyA was correct and the solution were free from impurities. The resolution of various molecular weight forms of HyA differed, but there was no difference in proton integration.

The molecular weights of HyA were confirmed by GPC using RI, LALS, RALS, and viscometer systems. The experimentally determined dn/dc value of HyA solutions investigated here was found to be 0.147 m/g, a value somewhat lower than previously reported dn/dc values, which range from 0.155 to 0.176 [32]. These variations in dn/dc values are most likely attributable to differences in the wavelength and solvent used (buffer containing 0.1 M NaCl in the present case). The weight-average molecular weights of HyAs obtained using GPC for each sample are given in Table 2. The standards, PEO 24 kDa and dextran 73 kDa, were confirmed to have molecular weights of 23,128 ± 573 and 72,060 ± 258 Da, respectively. The molecular weight of LMW-10, MMW-100, MMW-500, and HMW-1,500 were 13,241 ± 161 Da, 96,531 ± 1,167 Da, 512,657 ± 8,545 Da, and 1,249,500 ± 37,477 Da, respectively. The polydispersity (Mw/Mn) values of LMW-10, MMW-100, MMW-500, and HMW-1,500 were 1.376 ± 0.006, 1.389 ± 0.035, 1.304 ± 0.001, and 1.120 ± 0.044, respectively.

**Table. 2:** Molecular weights and polydispersity (Mw/Mn) of HyA measured by GPC

Samples		Mn (Da)	Mw (Da)	Mz (Da)	PDI
Standard materials	PEO 24k	22,652 ± 460	23,128 ± 573	23,430 ± 602	1.022 ± 0.005
	Dextran 73k	58,991 ± 190	72,060 ± 258	94,912 ± 1,169	1.170 ± 0.030
HyAs	LMW-10	9,013 ± 938	13,241 ± 161	16,951 ± 58	1.376 ± 0.006
	MMW-100	69,531 ± 882	96,531 ± 1,167	137,763 ± 1,217	1.389 ± 0.035
	MMW-500	365,273 ± 6,043	512,657 ± 8,545	748,347 ± 3,247	1.304 ± 0.001
	HMW-1,500	1,059,500 ± 4,950	1,249,500 ± 37,477	1,547,000 ± 56,569	1.120 ± 0.044

### Effects of HyA on the production of NO in macrophages

**Fig. 3:**
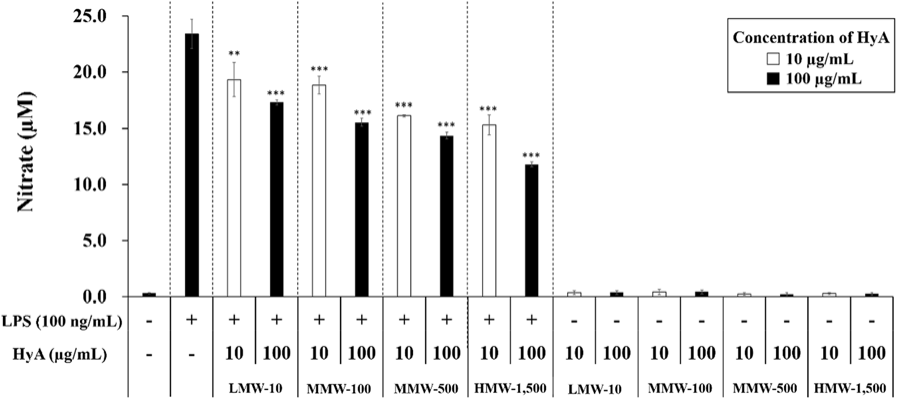
Effects of different molecular weights and concentrations of HyA on NO production in LPS-unstimulated and LPS-stimulated macrophages. LMW-10: low molecular weight of HyA (10 kDa); MMW-100: medium molecular weight of HyA (100 kDa); MMW-500: medium molecular weight of HyA (500 kDa); HMW-1,500: high molecular weight of HyA (1,500 kDa). Values are the mean ± S.D. of 9 experiments for *n* = 4. The statistical significance levels of the differences were set to *p < 0.05, **p < 0.01, and ***p < 0.001, HyA vs. LPS

NO is an ROS and free radical involved in many pathological and physiological processes [24]. It is also a major mediator of apoptosis and inflammatory processes that acts via complex mechanisms to play a role in nonspecific immunity associated with tissue injuries [33]. Accordingly, we evaluated the effects of different molecular weights and concentrations (10 and 100 µg/mL) of HyA on inflammatory responses in macrophages by assaying NO production. A shown in Fig. 3, we first found that 24-hour treatment with 100 ng/mL of LPS significantly increased NO production in macrophages (22.38 µM) compared with that in unstimulated macrophages (0.31 µM).

We also found that the various molecular weight forms of HyA alone had no effect on NO production in unstimulated macrophages. LPS-stimulated macrophages treated with 10 and 100 µg/mL of low molecular weight of HyA (LMW-10) showed NO production levels of 19.31 and 17.30 µM, respectively. For the higher molecular weight forms MMW-100, MMW-500, and HMW-1,500, HyA treatment of LPS-stimulated macrophages yielded NO production levels of 18.84, 16.15, and 15.31 µM, respectively, at 10 µg/mL, and 15.55, 14.37, and 11.79 µM, at 100 µg/mL.

### Distinct expression patterns of specific immune-related genes in macrophages treated with HyA

**Fig. 4:**
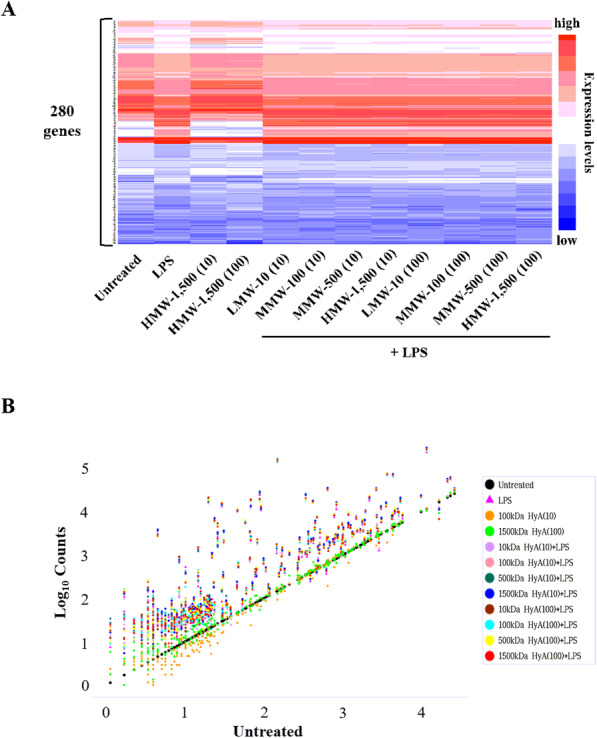
Gene expression analysis in LPS-stimulated macrophages treated with various molecular weights of HyA using NanoString. **A** Heatmap presenting significantly differential expression levels of the 280 immune genes in LPS-stimulated macrophages. The data are presented in matrix format. The rows represent each gene and the columns represent the treatment group. Expression levels have been normalized within columns for visualization. The red and blue colors reflect high and low expression levels, respectively. **B** A scatter plot showing differential expression levels of the 280 immune genes in 12 samples compared to the untreated (control) group. Untreated: control; LMW-10: low molecular weight of HyA (10 kDa); MMW-100: medium molecular weight of HyA (100 kDa); MMW-500: medium molecular weight of HyA (500 kDa); HMW-1,500: high molecular weight of HyA (1,500 kDa). (Concentration of HyA, unit: µg/mL)

**Fig. 5:**
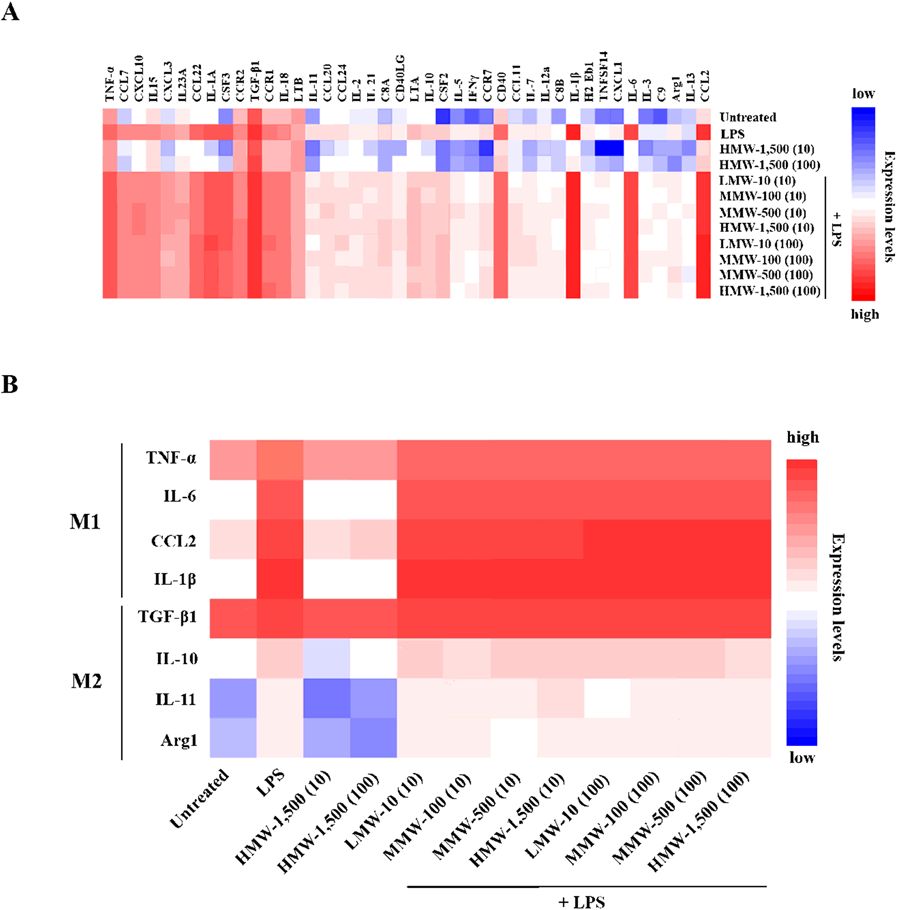
The heatmap of differentially expressed M1 and M2-related genes in LPS-stimulated macrophages treated with HyAs. **A** Heatmap showing NanoString analysis data from 42 (M1 and M2-related genes) of 280 immune gene expressions. **B** Data are shown for expression of 8 (M1: TNF-α, IL-6, CCL2, and IL-1β, M2: TGF-β1, IL-10, IL-11 and Arg1) of 42 genes. Color scale with red indicates highly expressed genes. (Concentration of HyA, unit: µg/mL)

Next, we studied the expression levels of 280 immune genes in LPS-stimulated macrophages treated with various molecular weights of HyA using NanoString technology (Fig. 4 A). The precision of a given method for relative measurements of gene expressions in LPS-stimulated macrophages is to be validated usually by evaluating similar results in both heatmap and scatter plot. For outcomes depicted as a heatmap, z-score data are applied to averaging gene levels, as shown in Fig. 4. The standard visualization technique used in scatter plots is a logarithmic transformation of data (Fig. 4 A) performed using nCounter (Fig. 4B), with each plot color-colored according to the immune genes. Scatter plots showed that compared with untreated macrophages (control), LPS-stimulated macrophages treated with various molecular weights and concentrations (10 and 100 µg/mL) of HyA showed over-expression of 42 immune genes (Fig. 5 A). Of these 42 immune genes in LPS-stimulated macrophages treated with different molecular weight forms of HyA, eight were associated with M1 or M2 macrophage-specific responses (Fig. 5B). In addition to the immune-specific genes (TNF-α, IL-6, IL-1β, TGF-β1, IL-10, IL-11 and CCL2), we found high expression levels of Arg1 in LPS-stimulated macrophages treated with various molecular weights forms of HyA. These eight genes were variously up- or down-regulated in in LPS-stimulated macrophages compared with controls in response to different molecular weights and concentrations of HyA.

With the exception of the highest molecular weight form, HMW-1,500, all HyA treatment groups showed up-regulated expression of the M1 associated genes, TNF-α, IL-6, CCL2 and IL-1β. IL-1β in particular was highly over-expressed in LPS-stimulated macrophages. Interestingly, all HyA treatment groups exhibited high levels of TGF-β1, which associated with the M2 macrophage phenotype. In contrast, expression levels of IL-10, IL-11 and Arg1 associated with M2 macrophages were lower relative to those genes associated with M1 macrophages (TNF‐α, IL-6, IL-1β, and CCL2). Overall, expression of genes associated with the M2 phenotype in LPS-stimulated macrophages, was lower in the HMW-1,500 treatment group than in other treatment groups.

### Effects of HyA on the expression of genes associated with the M1 phenotype in macrophages

We next quantitatively evaluated the effects of molecular weight and concentration of HyA on 4 genes expression of the four M1 macrophage-associated genes identified by nCounter with phase-TNF-α, IL-6, IL-1β, and CCL2-using RT-PCR.

**Fig. 6:**
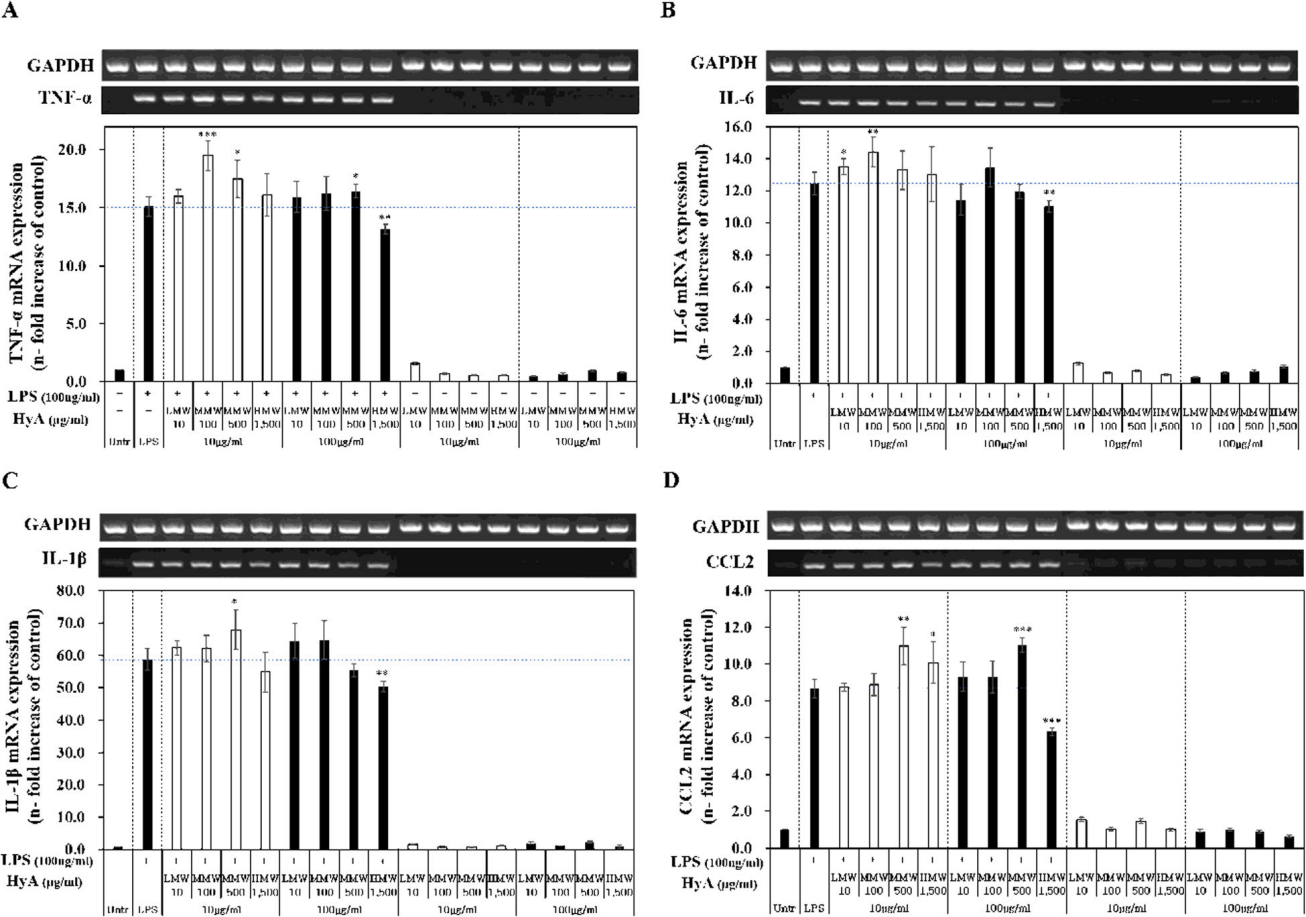
Gene expressions associated with M1 phenotype in LPS-unstimulated and LPS-stimulated macrophages treated with various molecular weights and concentrations of HyA. Macrophages were treated with various molecular weights of HyA (10 and 100 µg/mL) and 1 h later with LPS (100 ng/mL) for a 23- hour. GAPDH was used as a control. **A** TNF-α, **B** IL-6, **C** IL-1β and **D** CCL2 mRNA expressions were measured by RT-PCR. Data are expressed as the mean ± S.D. and are expressed as the relative fold increase. Untreated: control; LMW-10: low molecular weight of HyA (10 kDa); MMW-100: medium molecular weight of HyA (100 kDa); MMW-500: medium molecular weight of HyA (500 kDa); HMW-1,500: high molecular weight of HyA (1,500 kDa). Values are the mean ± S.D. of 9 experiments for n = 4. The statistical significance of the differences was set to *p < 0.05, **p < 0.01, and ***p < 0.001, HyA vs. LPS. (Concentration of HyA, blank: 10 µg/mL; filled: 100 µg/mL)

To this end, we treated unstimulated and LPS-stimulated macrophages with HyA at a high (100 µg/mL) and low (10 µg/mL) concentration. mRNA levels of each of these pro-inflammatory genes in macrophages treated with HyA are shown in Fig. 6.

TNF-α expression in LPS-stimulated macrophages was up‐regulated 15.1-fold (Fig. 6 A). In LPS-stimulated macrophages treated with 10 µg/mL of LMW-10, MMW-100, MMW-500, or HMW-1,500 groups, TNF‐α expression was up‐regulated by 16.0-, 19.5-, 17.5-, and 16.1-fold, respectively. Following treatment with 100 µg/mL of LMW-10, MMW-100, MMW-500, or HMW-1,500, TNF‐α expression in LPS-stimulated macrophages was up-regulated by 15.9-, 16.2-, 16.4- and 13.2-fold, respectively. This latter value for the HMW-1,500 group (13.2-fold) represents a 12.6 % suppression of TNF‐α expression relative to that in macrophages stimulates with LPS alone (15.1-fold).

As shown in Fig. 6B, IL-6 expression in LPS-stimulated macrophages was up‐regulated by 12.5-fold; this compared with 13.5-, 14.4-, 13.3-, and 13.0-fold increases in IL‐6 expression in LPS-stimulated macrophages treated with 10 µg/mL of LMW-10, MMW-100, MMW-500, or HMW-1,500, respectively. At a higher concentration (100 µg/mL), LMW-10, MMW-100, MMW-500, and HMW-1,500 treatment up-regulated IL‐6 expression in LPS-stimulated macrophages by 11.4-, 13.5-, 12.0-, and 11.0-fold, respectively, indicating a slight suppressive effect on IL‐6 expression at this higher concentration of HyA.

The overall pattern of IL-1β expression in LPS-stimulated macrophages (Fig. 6 C) was similar to that for IL6 expression (Fig. 6B), although levels of induction were higher for IL‐1β. Specifically, IL‐1β expression was up‐regulated by 58.8-fold in macrophages stimulate with LPS alone. In LPS-stimulated macrophages treated with 10 µg/mL of LMW-10, MMW-100, or MMW-500, IL‐1β expression was up‐regulated by 62.4-, 62.2-, and 68.0-fold, respectively. However, for LPS-stimulated macrophages treated with high molecular-weight form HyA (HMW-1,500) at a low concentration (10 µg/mL), IL‐1β expression was down‐regulated 54.8-fold in LPS-stimulated macrophages. Following treatment of LPS-stimulated macrophages with 100 µg/mL of LMW-10, MMW-100, MMW-500, or HMW-1,500, IL‐1β expression was also up‐regulated by 64.5-, 64.8-, 55.4-, and 50.4-fold, respectively. Interestingly, for MMW-500 at a high concentration (100 µg/mL), IL‐1β expression was slightly suppressed in LPS-stimulated macrophages. Treatment of LPS-stimulated macrophages with 100 µg/mL of HMW-1,500 also showed a suppression effect (14.2 %) on IL‐1β expression.

CCL2 expression in LPS-stimulated macrophages was up-regulated by 8.7-fold, as shown in Fig. 6D. In LPS-stimulated macrophages treated with 10 µg/mL of LMW-10, MMW-100, MMW-500, or HMW-1,500, CCL2 expression up‐regulated by 8.8-, 8.9-, 11.0-, and 10.1-fold, respectively. Following treatment of LPS-stimulated macrophages with LMW-10, MMW-100 and MMW-500 at the higher concentration of 100 µg/mL, CCL2 expression level was up‐regulated by 9.3-, 9.3-, and 11.1-fold. In contrast, LPS-stimulated macrophages with 100 µg/mL of HMW-1,500 exerted a 27.6 % suppressive effect on CCL2 expression. Thus, HMW-1,500 at a high concentration (100 µg/mL) significantly decreased expression of pro-inflammatory genes in LPS-stimulated macrophages. These results show that the molecular weight and concentration of HyA can significantly affect the pro-inflammatory response of LPS-unstimulated macrophages.

### Effects of HyA on the expression of genes associated with M2 phenotype in macrophages

**Fig. 7:**
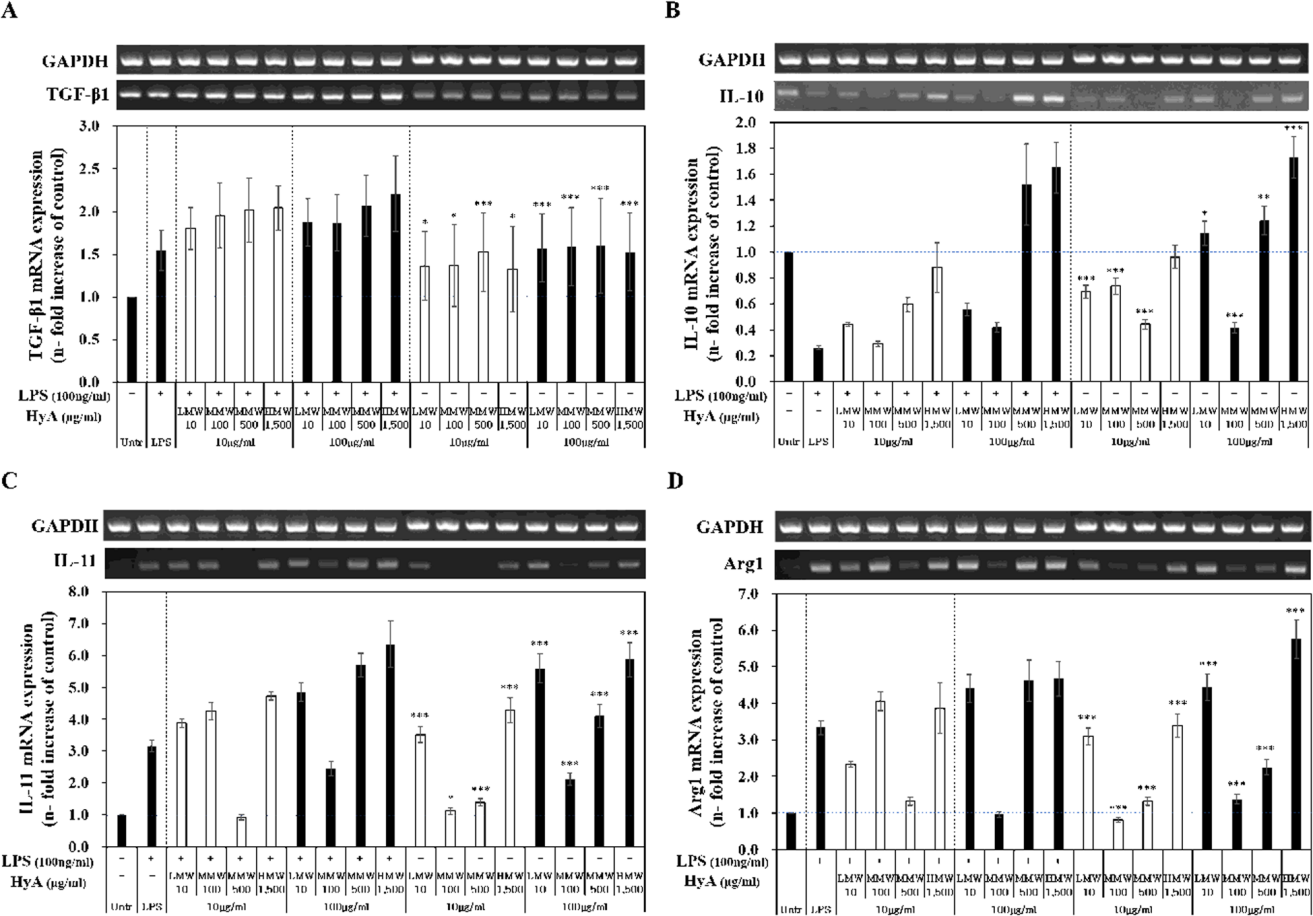
Gene expressions associated with M2 phenotype in LPS-unstimulated and LPS-stimulated macrophages treated with various molecular weights and concentrations of HyA. Macrophages were treated with various molecular weights of HyA (10 and 100 µg/mL) and 1 h later with LPS (100 ng/mL) for 23-hour. GAPDH was used as a control. **A** TGF-β1, **B** IL-10, **C** IL-11 and **D** Arg1 mRNA expressions were measured by RT-PCR. Data are expressed as the mean ± S.D. and are expressed as the relative fold increase. Untreated: control; LMW-10: low molecular weight of HyA (10 kDa); MMW-100: medium molecular weight of HyA (100 kDa); MMW-500: medium molecular weight of HyA (500 kDa); HMW-1,500: high molecular weight of HyA (1,500 kDa). Values are the mean ± S.D. of 9 experiments for *n* = 4. The statistical significance of the differences was set to *p < 0.05, **p < 0.01, and ***p < 0.001, HyA vs. Untreated. (Concentration of HyA, blank: 10 µg/mL; filled: 100 µg/mL)

Next, we assessed the effects of HyA molecular weight and concentration on the anti-inflammatory response of LPS-unstimulated and LPS-stimulated macrophages by quantitatively evaluating expression of the four M2 macrophage-associated genes identified by nCounter—TGF-β1, IL-10, IL-11, and Arg1—using RT-PCR, as described above for M1-associated genes. mRNA levels of each of these anti-inflammatory genes in macrophage treated with HyA are summarized in Fig. 7.

As shown in Fig. 7 A, TGF- β1 expression in macrophages stimulated with LPS alone was up-regulated 1.5-fold. In LPS-unstimulated macrophages treated with 10 µg/mL of LMW-10, MMW-100, MMW-500, or HMW-1,500 kDa, TGF-β1 expressions was slightly up-regulated by 1.3-, 1.3-, 1.4, and 1.4-fold, respectively. At the high concentration of 100 µg/mL, treatment with LMW-10, MMW-100, MMW-500, or HMW-1,500 kDa was also slightly up-regulated by 1.4-, 1.5-, 1.6, 1.4-fold, respectively. Interestingly, however, in all cases TGF-β1 expression in LPS-stimulated macrophages treated with either concentration of HyA increased with increases in the molecular weight of HyA. TGF-β1 expression was significantly up-regulated by 1.8-, 2.0-, 2.0, and 2.0-fold in LPS-stimulated macrophages treated with 10 µg/mL of LMW-10, MMW-100, MMW-500, or HMW-1,500 kDa, and showed a corresponding significant upregulation of 1.9-, 1.9-, 2.1, 2.5-fold following treatment with 100 µg/mL of LMW-10, MMW-100, MMW-500, or HMW-1,500 kDa. Although TGF-β1 expression in LPS-unstimulated macrophages was not significantly altered by any molecular weight form of HyA at either concentration, its expression in LPS-unstimulated macrophages was increased as the molecular weight of HyA increased, but was not affected by the concentration of HyA.

IL-10 expression levels in LPS-stimulated macrophages were significantly decreased by 0.3-fold (Fig. 7B). Although the effects of different molecular weight forms of HyA were not significant, there was a trend toward a difference in IL-10 expression levels with HyA concentration. Specifically, IL-10 expression in LPS-unstimulated and -stimulated macrophages treated with a low concentration (10 µg/mL) of different molecular weight forms of HyA was lower than that in controls. At a high concentration (100 µg/mL), IL-10 expression in LPS-unstimulated macrophages treated with LMW-10, MMW-500, or HMW-1,500 was slightly up-regulated by 1.1-, 1.2-, 1.7-fold, respectively; no change in IL-10 expression was observed in the MMW-100 group. IL-10 expression in LPS-stimulated macrophages treated with 100 µg/mL of MMW-500, or HMW-1,500 was up-regulated by 1.5- and 1.7-fold, respectively. Tends in IL-10 expression in macrophages treated with HyA with molecular weights great than 500 kDa were similar in LPS-unstimulated and -stimulated groups. IL-10 expression in LPS-unstimulated macrophages treated with low molecular-weight HyA (LMW-10) was similar to that observed following treatment with MMW-500. Although there was a tendency for IL-10 expression levels to differ according the concentration of HyA, this difference did not reach statistical significance.

IL-11 expression in LPS-unstimulated macrophages treated with 10 µg/mL of LMW-10, MMW-100, MMW-500, or HMW-1,500 was up-regulated by 3.5-, 1.1-, 1.4-, and 4.3-fold, respectively (Fig. 7 C). In LPS-unstimulated macrophages treated with 100 µg/mL of LMW-10, MMW-100, MMW-500, or HMW-1,500, IL-11 expression was up-regulated by 5.6-, 2.1-, 4.1-, and 5.9-fold, respectively. LPS-unstimulated macrophages treated with LMW-10 or HMW-1,500 showed a HyA-concentration–dependent increase IL-11 expression levels. For MMW-100 and MMW-500 treatment groups, however, IL-11 expression in LPS-unstimulated macrophages moved in opposite directions as function of concentration: for the MMW-100 group, IL-11 expression was up-regulated by 4.3-fold by treatment with 10 µg/mL and down-regulated by 2.5-fold by treatment with 100 µg/mL; for the MMW-500 group, IL-11 expression was down-regulated by 0.9-fold at 10 µg/mL up-regulated by 5.7-fold at 100 µg/mL.

As shown in Fig. 7D, Arg1 expressions in LPS-unstimulated and -stimulated macrophages treated with HyA showed a similar tendency to that of IL-11 (Fig. 7 C). Arg1 expression in LPS-unstimulated macrophages treated with a low concentration (10 µg/mL) of LMW-10, MMW-500, or HMW-1,500 was up-regulated by 3.1-, 1.3- and 3.4-fold, respectively; no changes in Arg1 expression were observed in the MMW-100 group. In LPS-unstimulated macrophages treated with 10 µg/mL of LMW-10, MMW-100, MMW-500, or HMW-1,500, Arg1 expression was up-regulated 4.4-, 1.4-, 2.3-, and 5.8-fold, respectively. We further found that LMW-10 or HMW-1,500 increased Arg1 expression levels in LPS-unstimulated macrophages in a concentration-dependent manner. The greatest Arg1 upregulation (4.9-fold) was observed for HMW-1,500 at a concentration of 10 µg/mL in LPS-stimulated macrophages.

IL-11 and Arg1 expression patterns in macrophages treated with small-molecular-weight form of HyA were similar to that of IL-10. Interestingly, at the high concentration (100 µg/mL), IL-11 and Arg1 expression in LPS-unstimulated and LPS-stimulated macrophages was highest in two groups: LMW-10, with the smallest molecular weight of 13 kDa, and HMW-1,500, with the largest molecular weight of 1,249 kDa. The expression behaviors of these genes in macrophages treated with HyA were not significantly dependent on LPS stimulation.

## Discussion

We examined the effects of various molecular weights of HyA in LPS-unstimulated and LPS-stimulated macrophages. This findings of this suggest that HyA has different effects with respect to its molecular weights. The data obtained show that the molecular weight of HyA was able to regulate inflammatory mediators differently, including cytokines and chemokines, in LPS-unstimulated and LPS-stimulated RAW 264.7 macrophages.

The structure and the molecular weights of each HyA were evaluated by ^1^ H NMR and GPC, respectively. The weight-average molecular weight of HyA obtained from GPC for each sample, LMW-10, MMW-100, MMW-500, and HMW-1,500, were 13,241 Da, 96,531 Da, 512,657 Da, and 1,249,500 Da, respectively. The Mw/Mn values of LMW-10, MMW-100, MMW-500, and HMW-1,500 were 1.376, 1.389, 1.304, and 1.120, respectively. There was no difference in protons integration, as shown in ^1^ H NMR in Fig. 1. We confirmed the chemical structure and the molecular weight of HyA without any impurity.

NO is endogenously biosynthesized from L-arginine in a reaction catalyzed by different NO synthase (NOS) enzymes. However, the enzyme mainly acts as NO in inflammatory processes as the inducible nitric oxide synthase (iNOS) [33, 34]. NO must be induced by particular cytokines such as IL-6, TNF-α or LPS, and released by iNOS is not mostly expressed in resting cells [34, 35].

In a NO assay in LPS-stimulated macrophages, HyA showed dependent effects on macrophages according to its molecular weight [10]. Low molecular weight of HyA (50 kDa) significantly increase iNOS levels in LPS-stimulated chondrocytes. Middle-molecular-weight HyA (1,000 kDa) exerts no significant effect on iNOS in LPS-stimulated chondrocytes. High molecular weight of HyA (5,000 kDa) significantly reduces the LPS-induced increment in iNOS. No effect was exerted in LPS-unstimulated chondrocytes [36]. Therefore, in this study, we evaluated the effects of various molecular weights of HyA on the ability to inhibit inflammation. Macrophages were treated with various molecular weight of HyA (10 and 100 µg/mL) and 1 h later with LPS to mimic an inflammatory response. After 24 h, HMW-1,500 could suppress NO production better than LMW-10 in LPS-stimulated macrophages (Fig. 3). NO production in LPS-stimulated macrophages decreased with increase in not only concentration of HyA treated, but in molecular size of HyA. At a high concentration of 100 µg/mL, the largest molecular weight of HyA, HMW-1,500, reduced NO production in LPS-stimulated macrophages to about 45 %. Campo et al. reported that the interaction of high molecular weight HyA of 5,000 kDa and TLR-4 prevented LPS action on cells [38]. But, our result showed that HyA with a molecular weight of more than 1,250 kDa could sufficiently reduce the NO production in LPS-stimulated macrophages.

Depending on their cytokine secretion pattern and cellular phenotype, macrophages are able to either suppress or mediate inflammatory responses. Macrophages from M1 to M2 render a considerable benefit in relation to autoimmune diseases and in, the regeneration of injured tissues. They can also serve as a treatment for inflammation. It has been shown that the molecular weights of polymers have the ability to raise different biological responses [1, 37]. In this regard, we investigated the effects of various molecular weights of HyA on specific immune gene expression levels in LPS-unstimulated and LPS-stimulated macrophages (Figs. 6 and 7). LPS is able to produce many inflammatory mediators, such as IL-6 and TNF-α and to mediate acute inflammation as a potent macrophage activator [38, 39]. But, macrophages simultaneously treated with LPS and HyAs show contrary effect with corresponding decreasing TNF-α expression levels [40]. Others reported that high molecular weight of HyA significantly reduced TNF-α and IL-1β expression levels of LPS-stimulated cells [36].

With regard to IL-10, it demonstrated significant up-regulation in a treatment with high molecular weight of HyA in unstimulated macrophages compared to the other conditions examined. IL-10 is an anti-inflammatory cytokine commonly produced by M2 phase [41, 42]. Arg1 expression is a signature of M2 phase polarization. Arg1 expression was significantly up-regulated in macrophages treated with HyA (3,000 kDa) compared to LPS-unstimulated macrophages. This suggests movement to an alternatively activated state. It was revealed that the highest Arg1 expression levels was acquired in LPS-stimulated macrophages treated with high molecular weight of HyA [11]. In our study, it was confirmed that the expressions of the pro-inflammatory genes such as TNF-α, IL-6, IL-1β and CCL2 decreased in LPS-stimulated macrophages treated with high molecular weight HyA above 1,250 kDa (HMW-1,500), identical to the findings of earlier studies.

Much research have been studied on the pro-inflammatory immune response of hyaluronic acid, but little have been studied on the anti-inflammatory immune response. From this study, we searched specific immune genes, TGF-β1, IL-10, IL-11, and Arg1 at high expression levels in LPS-stimulated macrophages treated with various molecular weights of HyA at a high and a low concentrations.

For anti-inflammatory genes, the specific M2 associated genes were up-regulated or down-regulated in the different molecular weights and concentrations of HyA in macrophages compared to the control. The expression of IL-10 was highest in LPS-stimulated macrophages treated with HMW-1,500 at a high concentration of 100 µg/mL. The expressions of IL-10, associated with M2 phase in LPS-stimulated macrophages was significantly influenced by the molecular weight of HyA, and was hardly affected by the concentration of HyA.

The expressions of IL-11 and Arg1 in macrophages treated with small molecular weight of HyA was similar to that of IL-10. The express behaviors of these genes in macrophages treated with HyA were not significantly dependent of LPS stimulating. The expression of IL-11 and Arg1 were highest in LPS-stimulated macrophages treated with HMW-1,500 at a low concentration of 10 µg/mL. At a high concentration of 100 µg/mL, interestingly, the expressions of IL-11 and Arg1 in LPS-unstimulated and LPS-stimulated macrophages were highest in the two groups: the smallest molecular weight of 13 kDa (LMW-10), and the largest molecular weight of 1,250 kDa (HMW-1,500) in this study.

In previous studies, the effects were demonstrated mostly with high molecular weight of HyA, which was able in LPS-stimulated cells to reduce not only TRAF-6 and MyD88 as well as TLR-4 receptor expression levels but also NF-κB activation and the increase in pro-inflammatory cytokines [38]. However, low molecular weight of HyA exerted only a slight increase in inflammatory mediators in LPS-unstimulated cells, whereas it enhanced TRAF-6 and MyD88 as well as TLR-4 receptor expression levels along with NF-κB activation and pro-inflammatory cytokine levels, in LPS-stimulated cells compared to cells treated only with LPS [43, 44]. Medium molecular weight of HyA did not show any activity on inflammatory mediators in untreated cells. It was unable to affect any considered parameter in LPS-stimulated cells. Thus, the positive regulatory effect exerted by high molecular weight of HyA on all of the parameters considered here could be due to the binding of TLR-4, thereby preventing receptor stimulation by specific ligands [45, 46]. This is in contrast with the low molecular weight HyA, which instead had a stimulatory effect by enhancing LPS stimulation. We could confirm the signal pathway in earlier work [38]. That is, TNF-α and IL-6 were increased in LPS-stimulated macrophages treated with medium molecular weight HyA of 97 kDa (MMW-100). IL-1β and CCL2 were also increased in LPS-stimulated macrophages treated with MMW-100. In contrast, it was confirmed that the pro-inflammatory response was reduced in LPS-macrophages treated with high molecular weight HyA of above 1,250 kDa (HMW-1,500) (Fig. 6). IL-10, IL-11, and Arg1 except TGF-β1 were decreased in LPS-stimulated macrophages treated with medium molecular weight HyA of 97 kDa, and 513 kDa, MMW-100 and MMW-500, respectively. Thus, high molecular weight HyA of above 1,250 kDa reduced the pro-inflammatory response in LPS-stimulated macrophages, and promoted the anti-inflammatory response in LPS-stimulated macrophages.

## Conclusions

In this study, the molecular weight of HyA was accurately measured using GPC with light scattering, demonstrating well controlled molecular weight distributions of HyA samples employed. NO production decrease in a concentration-dependent manner in LPS-stimulated macrophages treated with HyA. High molecular weight HyA of above 1,250 kDa (HMW-1,500) at a high concentration of 100 µg/mL inhibited NO production most in LPS-stimulated macrophages. We also found to be over-expressed specific immune genes (TNF-α, IL-6, IL-1β, TGF-β1, IL-10, IL-11, CCL2, and Arg1) in LPS-stimulated macrophages treated with various molecular weights of HyA by using Nanostring technology via a heatmap and a scatter plot. The gene expressions, analyzed by RT-PCR, were evaluated that LPS-stimulated macrophages treated with high molecular weight HyA of above 1,250 kDa were down-regulated in terms of the expressions of TNF‐α, IL-6, CCL2 and IL-1β. However, expression levels of IL-10, IL-11 and Arg1 were highest, except for TGF-β1 in LPS-unstimulated macrophages treated with high molecular weight HyA of above 1,250 kDa. These findings suggest that high molecular weight HyA of above 1,250 kDa can induce M2 phase polarization in unstimulated-macrophages without LPS. We concluded that various molecular weights of HyA can regulate immune responses in M1 and M2 phase, and that high molecular weight HyA of above 1,250 kDa at a high concentration of 100 µg/mL suppresses the immune response.

## Data Availability

Not applicable.
